# The effect of probiotics on functional constipation in adults: A randomized, double-blind controlled trial

**DOI:** 10.1097/MD.0000000000031185

**Published:** 2022-10-28

**Authors:** Fabiana Cristina Rosa Mitelmão, Karin Häckel, Cristiane de Cássia Bergamaschi, Marli Gerenutti, Marcus Tolentino Silva, Victor Manuel Balcão, Marta Maria Duarte Carvalho Vila

**Affiliations:** a PhageLab - Laboratory of Biofilms and Bacteriophages, University of Sorocaba, Sorocaba/SP, Brazil; b Clinic of Gastroenterology Dr Karin Häckel, Sorocaba/SP, Brazil; c Pontifical Catholic University of São Paulo (PUC-SP), Sorocaba/SP, Brazil; d Department of Biology and CESAM, University of Aveiro, Campus Universitário DE Santiago, Aveiro, Portugal.

**Keywords:** *Bifidobacterium*, clinical trial, functional constipation, *Lactobacillus*

## Abstract

Two formulations were developed in the form of an oral sachet containing probiotics, and their efficacy and safety were evaluated in adults with functional constipation.

**Methods:**

One formulation with *Lactobacillus acidophilus, Bifidobacterium bifidum and Lactobacillus rhamnosus* (3 billion Colony Forming Units - CFU); and another with *Lactobacillus acidophilus, Bifidobacterium bifidum, Lactobacillus rhamnosus, Lactobacillus paracasei, Bifidobacterium longum, Bifidobacterium lactis, Lactobacillus casei, Bifidobacterium animallis* (8 billion CFU). The participants were randomized in a 3-arm parallel study and one oral sachet was auto-administered once a day for 30 days.

**Results:**

Primary outcomes were improvement in increasing the frequency of weekly bowel movements and improvement in stool quality. Secondary outcomes were number of adverse events. In the first week one observed an increase in stool frequency and in the quality of stools, showing an improvement in constipation. No statistically significant differences were observed between the three treatment groups in relation to these outcomes (*P *≥ .05). Only one adverse event was observed in a patient of group 2, related to abdominal pain.

**Conclusion:**

The two probiotic cocktails were effective in improving the symptoms of functional constipation, by increasing both the weekly frequency of evacuation and stool quality, and were deemed safe. Clinicaltrials.gov number: NCT04437147.

## 1. Introduction

Of the gastrointestinal disorders, constipation is one of the most reported conditions in clinical practice.^[1,2]^ Constipation affects between 15% and 20% of adult humans, of which 33% are over 60 year-old, with predominance in women. The medical history of patients with constipation should be analyzed together with parameters such as fecal consistency, defecating frequency, effort needed to defecate, feeling of incomplete evacuation, abdominal pain and discomfort, use of laxatives, surgical history, comorbidities, lifestyle and work activity.^[3]^ Functional constipation is based on symptoms of nonorganic origin and diagnosed by the diagnostic criteria of Rome IV.^[4]^ The Bristol Stool Scale can help patients assess and describe aspects of their stools, facilitating the recognition of constipation severity.^[5]^ Intestinal constipation negatively impacts the quality of life and can lead to significant costs in the search for treatments and purchase of laxatives.^[1,6]^ The treatment of constipation is a challenge in the sense that osmotic, stimulating, irritating, and prokinetic laxatives are usually used.^[2]^ However, it appears that up to 47% of the patients are not completely satisfied due to inconsistent response to laxatives and concerns about their safety, adverse effects, taste, inconvenience, and cost.^[7]^

Currently, it is known that there is an important interaction between microbes and intestinal physiology. Therefore, probiotics have been used to treat many intestinal disorders,^[8]^ such as infectious diarrhea, diarrhea associated with antibiotics, diarrhea associated with *Clostridium difficile*, hepatic encephalopathy, ulcerative colitis, irritable bowel syndrome, functional gastrointestinal disorders, necrotizing enterocolitis, and functional constipation.^[9–11]^

Although the human gut microbiome comprises >400 bacterial species, evidence has shown that a decrease in the population of *Bifidobacterium* and *Lactobacillus* in adults can result in intestinal constipation.^[12–14]^ Consequently, the probiotics used in humans for the treatment of constipation are more often of the species *Lactobacillus* and *Bifidobacterium. Lactobacillus* and *Bifidobacterium* can shorten the migratory myoelectric complex period and accelerate small intestine transit, partly due to increased release of serotonin (5-HT) which has promotility effects.^[8]^

Clinical trials evaluating different strains of *Lactobacillus* and *Bifidobacterium* in the treatment of intestinal constipation have observed promising results,^[15–19]^ conclusions also supported by systematic reviews on the topic.^[20]^ However, there is no consensus on both the types of probiotic strains and their dosages for the treatment of constipation.^[12,21]^ Strain selection is an important step in the production of a probiotic. Probiotics should have a beneficial effect on the host and remain viable throughout the product lifetime.^[22]^

The clinical trial reported herein evaluated the efficacy and safety of 2 different probiotic cocktails when compared to a conventional fiber treatment. One of the probiotic cocktails integrated 4 strains of *Lactobacilli* (*Lactobacillus acidophilus* [LA], *Lactobacillus rhamnosus* [LR], *Lactobacillus paracasei* [LP], and LC) and 4 strains of *Bifidobacteria* (*Bifidobacterium bifidum* [BB], *Bifidobacterium longum*, *Bifidobacterium lactis* and *Bifidobacterium animalis*). The other probiotic cocktail integrated 2 strains of *Lactobacilli* (LA and LR) and 1 strain of *Bifidobacteria* (BB). Hence, this study aimed at confirming whether the dosage of probiotic strains increased the weekly frequency of bowel movements and the quality of stools, improving intestinal functional constipation in human subjects.

## 2. Methods

### 2.1. Study design and setting

The study entertained herein consisted in a single-center, randomized, double-blind and controlled clinical trial, registered in ClinicalTrials.gov (Identifier: NCT04437147; https://clinicaltrials.gov/ct2/show/NCT04437147). This study followed the CONSORT 2010 checklist of information to include when reporting a randomized trial (available at http://www.consort-statement.org/media/default/downloads/CONSORT%202010%20Checklist.pdf), and other information is available in the published protocol of this protocol.^[23]^

The parallel clinical trial worked with the hypothesis that the groups that received probiotics had a greater increase in weekly frequency of bowel movements and stool quality, being therefore more effective than the group that received the conventional fiber treatment. During a 4-week timeframe, 153 patients with functional constipation (51 patients per group) were treated as follows: the first group received a probiotic cocktail containing 3 billion colony forming units (CFU) of mixed strains of probiotic bacteria per sachet, the second group was treated with 8 billion CFU of mixed strains of probiotic bacteria per sachet, and the third group was treated with the conventional fiber treatment for constipation (composed by prebiotic fibers, vitamins, and minerals).

The study was carried out single-center, at Dr Karin Häckel’s Gastroenterology Clinic, located in Sorocaba, State of São Paulo, Brazil. The recruitment of patients for this study was carried out through a collaborative effort between the University of Sorocaba and Dr Karin Häckel at the Clinic of Gastroenterology.

For the dissemination of the study, digital platforms were used, and letters were distributed via e-mail and *in Campus*, via mobile phone text messages, and social networks were also explored to promote the study. Recruitment of participants was carried out until November 30th, 2020.

After verbal and written clarification of the study, the participants that agreed to enter in the study signed the Informed Consent Form already approved by the Ethics Committee of the University of Sorocaba. All authors had access to the study data and reviewed and approved the final version of the manuscript.

### 2.2. Eligibility criteria

#### 2.2.1. Inclusion criteria

Eligible patients for the study were adults aged 20 to 80 year-old with clinical diagnosis of functional constipation according to the Rome IV Consensus.

The Rome IV Consensus defines functional constipation as a dysfunction that manifests itself as difficult, infrequent, and incomplete bowel movements. Constipation must have started 6 months earlier and become more frequent in the past 3 months, including 2 or more of the following characteristics: involving <25% of bowel movements (straining, hardened resistance - Bristol scale 1–2), feeling of incomplete evacuation, a sensation of anorectal obstruction, digital maneuvers to facilitate the removal of fecal content, <3 spontaneous evacuations/week and need for laxatives.^[4]^ Participants entered the study only after granting written authorization.

#### 2.2.2. Exclusion criteria

The exclusion criteria were the presence of gastrointestinal diseases, use of antibiotics or dietary supplements containing probiotics or prebiotics in the last 15 days, and pregnancy.

### 2.3. Interventions

Participants were instructed to store the sachets at room temperature and to take a sachet before breakfast, by dissolving the contents of a sachet in 150 mL of water. To improve treatment adherence, phone calls and/or messages were sent to verify that participants were following the correct protocol and working as planned.

During the clinical trial, the use of laxatives was prohibited. The sachets were auto-administered during a timeframe of 30 days for all participants. The study consisted of 3 parallel arms:

1. **Active comparator:** 3 billion CFU of probiotic bacteria (3 × 109 CFU per sachet)–strains LA 02 ID 1688 (1 billion CFU), BB 01 ID 1722 (1 billion CFU), LR 04 ID 1132 (1 billion CFU), vitamin C (ascorbic acid) 45 mg, vitamin B1 (thiamin) 1.1 mg, vitamin B2 (riboflavin) 1.1 mg, vitamin D3 (cholecalciferol) 40.000.000 IU/g (34 µg), magnesium hydroxide 0.3 g, calcium carbonate 0.5 g, natural vanilla flavor powder 0.03 g, fructooligosaccharides (FOS) up to 3 g: one sachet/day for 30 days.
2. **Active comparator:** 8 billion CFU of probiotic bacteria (8 × 109 CFU per sachet)–strains LPC 00 ID 1076 (1 billion CFU); *Bifidobacterium longum* (BL) 03 ID 1152 (1 billion CFU); *Bifidobacterium lactis* (BS) 01 ID 1195 (1 billion CFU); *Lactobacillus casei* (LC) 03 ID 1872 (1 billion CFU); *Bifidobacterium animalis* THT 010803 (1 billion CFU); LA 02 ID 1688 (1 billion CFU), BB 01 ID 1722 (1 billion CFU), LR 04 ID 1132 (1 billion CFU), vitamin C (ascorbic acid) 45 mg, vitamin B1 (thiamin) 1.1 mg, vitamin B2 (riboflavin) 1.1 mg, vitamin D3 (cholecalciferol) 40.000.000 IU/g (34 µg), magnesium hydroxide 0.3 g, calcium carbonate 0.5 g, natural vanilla flavor powder 0.03 g, FOS up to 3 g: one sachet/day for 30 days.
3. **Conventional fiber treatment:** vitamin C (ascorbic acid) 45 mg, vitamin B1 (thiamin) 1.1 mg, vitamin B2 (riboflavin) 1.1 mg, vitamin D3 (cholecalciferol) 40.000.000 IU/g (34 µg), magnesium hydroxide 0.3 g, calcium carbonate 0.5 g, natural vanilla flavor powder 0.03 g, FOS up to 3 g: one sachet/day for 30 days.

The follow-up of patients was of 1 week after the end of the use of probiotics.

### 2.4. Measured outcomes

#### 2.4.1. Primary outcomes

The changes in bowel frequency (movements and quality of the stools) were annotated in a Table that the patients filled in with information regarding the daily frequency of evacuation and type of stools (on a scale of 1–7 in the Bristol scale, or if there was no evacuation). The stool form was collected using the Bristol stool form scale (BSFS), a simple tool to estimate intestinal transit time. The BSFS classifies stools into 7 categories, including type 1 (separate hard lumps such as walnuts); type 2 (sausage-shaped but irregular); type 3 (like sausage but with cracks on the surface); type 4 (such as sausage or snake, smooth and soft); type 5 (smooth bubbles with sharp edges); type 6 (fluffy pieces with jagged edges, pasty stools); type 7 (aqueous, in solid pieces).^[5]^ These stool types are categorized into slow transit (types 1 and 2), normal transit (types 3–5), and rapid transit (types 6 and 7).

The metric of analysis was the comparison between 0 and 30 days, considering that the number of effective bowel movements over 4 times a week is an effective value for the treatment and the quality of the stools from types 3 to 5.

#### 2.4.2. Secondary outcomes

Adverse events are undesirable signs or symptoms that occur during the study and occurrences that may or may not be causally related to the treatment. All adverse events considered possible or likely related to the test product were registered in the patient form.

Serious adverse events are defined as fatal, life-threatening, disabling, resulting in hospitalization or prolonged stay, or resulting in malformation, whether related to the product under test or otherwise. According to previous studies, probiotics are safe and any serious adverse event that could be related to the products under test would be considered unexpected. Any unexpected serious adverse events were to be reported to the physician. Any serious adverse events that could be related to the product under test would immediately lead to the discontinuation of the product under test.^[13]^ The number of patients and of adverse events and serious adverse events were duly recorded.

### 2.5. Sample size and recruitment

**Figure 1.**
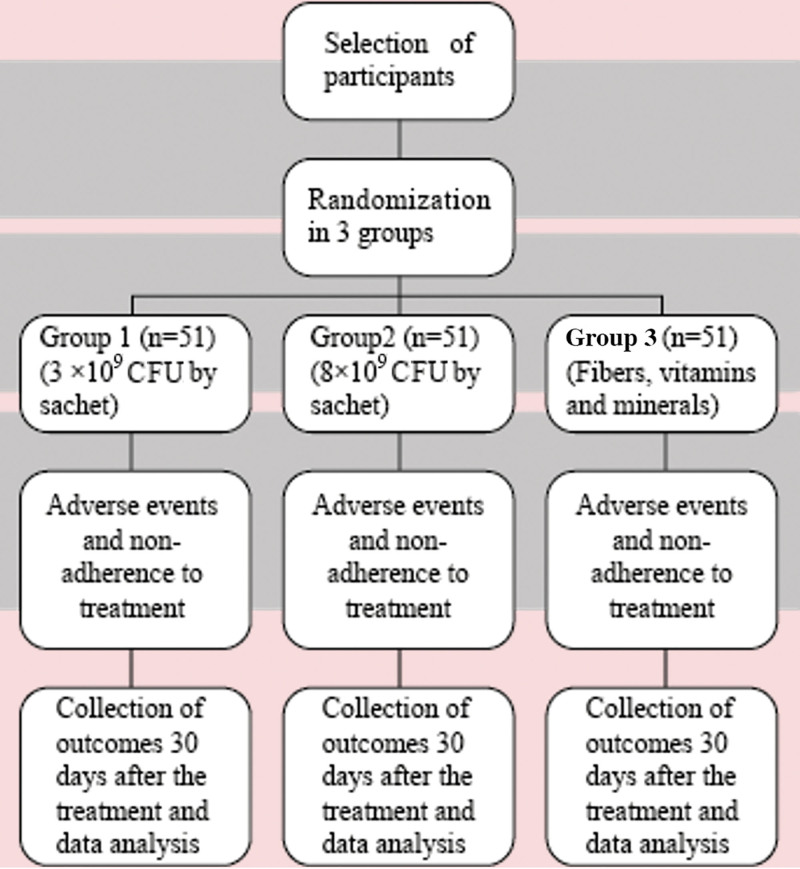
Research group enrollment in the trial and treatment schedule. BSFS = Bristol stool form scale, CFU = colony forming unit.

The sample size was calculated based on two relative means and their respective standard deviations related to the weekly increase in bowel movements and stool consistency considering the data reported by Del Piano et al (2010).^[15]^ The correlation was established using epidemiological statistics available on the OpenEpi website (OPENEPI, 2013). As a 15% dropout rate is expected, 153 participants were included in the study, aiming to reach the completion of at least 132 participants. The participants that entered the study and the treatment schedule are displayed in Figure 1.

### 2.6. Randomization and allocation concealment for treatment

Randomization was performed by Random Allocation Software. Participants were equally stratified into 3 groups, each with blocks of 9 participants.

After confirming the eligibility and reading/signing the Informed Consent Form, the participants received an identical sealed opaque envelope with the randomized sequential number, by one of the researchers Fabiana Cristina Rosa Mitelmão (FCRM). The packaging differed only in the number of manufacturing batches. All groups received identical sachets (same flavor, color and packaging), with no possibility of differentiating one batch from another.

The physician Karin Häckel (KH) selected each participant according to the batch number corresponding to the randomization. Eligible participants were allocated (1:1:1 to receive treatment, for 4 weeks, with the probiotic supplement containing 3 × 109 CFU per sachet, 8 × 109 CFU per sachet, or conventional fiber treatment). Study participants and the investigator KH that provided the treatment and collected the outcomes were blinded.

To promote participant adherence to the study and complete follow-up, the text message or call was sent out after 15 days to find out how the treatment was and whether there was discontinuation or deviation from the intervention protocol. The following questions were asked: Why did you not continue the treatment? Have you had any adverse events, if any? Participants were instructed to fill in the correct form if any adverse events occurred and what kind of stools and daily rate of evacuation.

Participants could also be removed from the study if the treatment was interrupted in any way for any reason, whether due to forgetfulness or undue intestinal disconfort. Unmasking and revealing the intervention during the study was allowed if there was a serious adverse event reported, and the physician would investigate if the product was actually the cause. Participants were allowed to withdraw from the study at any time at their own request or be withdrawn at any time at the investigator’s discretion for safety reasons.

### 2.8. Data treatment and record keeping

Data from the participant’s medical records were entered into a Microsoft Excel spreadsheet and the medical records were stored in a safe place for proof of the study. The data collection form was used to record patient data from all participants and completed by the researcher FCRM, who also registered the data in a Microsoft Excel spreadsheet. The results measured were verified, in duplicate, to ensure their quality.

### 2.9. Data analysis

The statistical analysis of variance test was used to compare BSFS scores between groups whereas the paired *t* test was used to to compare BSFS scores within groups at different times. The level of statistical significance was set at *P* < .05. Statistical analysis was performed using the software STATA v.14.2. (https://www.stata.com/stata14/) (StataCorp LLC, Texas TX).

### 2.10. Ethics and disclosure

The project was filed under the number CEP-Single CAAE: 84003418.9.0000.5500 and was approved on 09/17/2018. Informed and signed consents were declared at the clinic participating in the research by the outcome evaluators KH. Personal information about the participants was collected and kept confidential before, during, and after the end of the clinical trial by only one of the researchers KH, which was delivered to the researcher responsible for it FCRM. The researchers stated that they had no financial interest whatsoever in this clinical study. All authors had access to the study data and reviewed and approved the final manuscript.

## 3. Results

**Figure 2.**
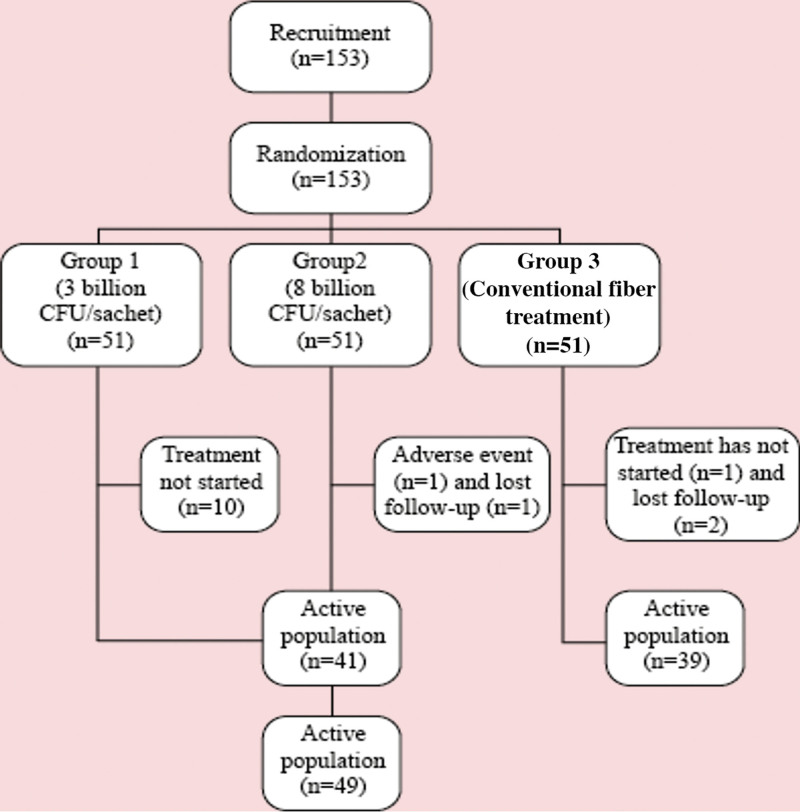
Distribution of patients in the study groups.

A total of 153 participants were selected and 132 were enrolled in the study after clarification of the clinical trial and signature of the informed consent form. Following randomization, 41 participants were treated with 3 billion CFU/sachet (Group 1), 49 participants were treated with 8 billion CFU/sachet (Group 2) and 39 participants were treated with the conventional fiber treatment (Group 3). A non-pharmacological treatment is the first-line management of constipation involving the use of fibers.^[24]^ Figure 2 shows the distribution of participants in the study groups. Table 1 shows the characteristics of the groups in which women with average ages of 39.9 to 43.1 year-old predominated.

**Table 1:** Characteristics of the participants entering the study.

	Group 1	Group 2	Group 3	1 × 2	1 × 3	2 × 3	1 × 2 × 3
Parameters	Average ± σ	Average ±σ	Average ±σ	*P* value^1^	*P* value^2^	*P* value^3^	*P* value^4^
Women (%)	87.8 (n = 36)	98.0 (n = 50)	97.5 (n = 39)	.085	.201	1.000	.086
Age	39.9 ± 12.6 (41)	43.1 ± 16.1 (51)	40.9 ± 14.7 (40)	.005	.023	.588	.013

1: *P* value of the two-tailed *t*-student test to identify the differences between groups 1 and 2 at weeks 0, 1, 2, 3, 4.

2: *P* value of the two-tailed *t*-student test to identify the differences between groups 1 and 3 at weeks 0, 1, 2, 3, 4.

3: *P* value of the two-tailed *t*-student test to identify the differences between groups 2 and 3 at weeks 0, 1, 2, 3, 4.

4: *P* value of the analysis of variance to identify the differences between groups 1, 2 and 3 in weeks 0, 1,2,3,4.

Group 1 = Formula with 3 billion CFU/sachet, Group 2 = Formula with 8 billion CFU/sachet, Group 3 = conventional fiber treatment, CFU = colony-forming units, 1 × 2 = group 1 compared to group 2, 1 × 3 = group 1 compared to group 3, 2 × 3 = group 2 compared to group 3, 1 × 2 × 3 = group 1 compared to group 2 and group 3, σ = standard deviation.

Table 2 presents the results obtained in relation to the frequency of bowel movements and the quality of stools. The analysis metric was a comparison between week zero until week 4 of the study. No statistically significant differences were observed between the three treatment groups in relation to the frequency of weekly bowel movements, which increased in all three groups as well as in improving stool quality (*P *≥ .05). The single adverse event that was reported in only one participant in Group 2, was related to abdominal pain.

**Table 2:** Distribution of participants in the study groups according to the CONSORT diagram, and demographic characteristics of the three groups with the intention to treat.

	Group 1 36 women 5 men			Group 2 50 women 1 man			Group 3 39 women 1 man			1 × 2	1 × 3	2 × 3	1 × 2 × 3
Parameter	Average ± σ	n	*P* value¹	Average ±σ	n	*P* value²	Average ±σ	n	*P* value³	*P* value^4^	*P* value^5^	*P* value^6^	*P* value^7^
Weekly number of evacuations													
Week 0	1.9 ± 0.8	41		1.8 ± 0.8	51		1.7 ± 0.8	40		.481	.149	.413	.353
Week 1	4.9 ± 2.5	41	<.001	4.7 ± 2.0	51	<.001	6.4 ± 7.7	40	<.001	.818	.224	.141	.187
Week 2	5.8 ± 2.7	41	<.001	5.1 ± 2.0	50	<.001	5.9 ± 2.9	39	<.001	.119	.946	.118	.217
Week 3	5.5 ± 2.6	41	<.001	5.0 ± 1.8	49	<.001	6.1 ± 2.4	39	<.001	.262	.278	.011	.066
Week 4	5.2 ± 1.8	41	<.001	5.1 ± 2.0	49	<.001	5.5 ± 2.3	39	<.001	.766	.645	.465	.744
Stool quality according to the Bristol Scale*													
Week 0	1.9 ± 0.8	41		1.8 ± 0.8	51		1.7 ± 0.8	40		.481	.149	.413	.353
Week 1	3.0 ± 1.2	41	<.001	2.9 ± 1.1	51	<.001	2.7 ± 0.9	40	.004	.739	.253	.366	.499
Week 2	3.2 ± 1.1	41	<.001	2.9 ± 1.1	50	<.001	3.2 ± 0.9	39	<.001	.291	.928	.294	.449
Week 3	3.1 ± 1.1	41	<.001	3.4 ± 1.1	49	<.001	3.4 ± 1.1	39	<.001	.219	.250	.966	.395
Week 4	3.6 ± 1.1	41	<.001	3.3 ± 0.9	49	<.001	3.3 ± 1.0	39	<.001	.278	.310	.974	.469

1: *P* value of the two-tailed paired *t* student test to identify differences between weeks 1, 2, 3, 4 with week 0 in group 1.

2: *P* value of the two-tailed paired *t* student test to identify differences between weeks 1, 2, 3, 4 with week 0 in group 2.

3: *P* value of the two-tailed paired *t* student test to identify differences between weeks 1, 2, 3, 4 with week 0 in group 3.

4: *P* value of the two-tailed paired *t* student test to identify the differences between groups 1 and 2 at weeks 0, 1, 2, 3, 4.

5: *P* value of the two-tailed paired *t* student test to identify the differences between groups 1 and 3 at weeks 0, 1, 2, 3, 4.

6: *P* value of the two-tailed paired *t* student test to identify the differences between groups 2 and 3 at weeks 0, 1, 2, 3, 4.

7: *P* value of the analysis of variance to identify the differences between groups 1, 2, and 3 in weeks 0, 1, 2, 3, 4.

Group 1 = formula with 3 billion CFU/sachet, Group 2 = formula with 8 billion CFU/sachet, Group 3 = conventional fiber treatment, CFU = colony-forming units.

* Stool quality according to the Bristol Scale according to Lewis and Heaton (1997).

## 4. Discussion

The study entertained herein demonstrated that treatment with a new probiotic cocktail of either 3 LAB strains (LA 02, BB 01, LR 04) or 8 LAB strains (LA 02, BB 01, LR 04, LPC 00, BL 03, BS 01, LC 03, THT 010803), ingested once a day, for 30 days, increased the weekly frequency of evacuation and the quality of stools in patients, who reported improvement in functional intestinal constipation. In addition, the probiotic mixtures showed favorable safety profiles, as evidenced with the report of only one adverse event that was not considered a serious event.

Therefore, combination of different LAB strains did not have an impact in either their efficacy and safety, compared to the conventional fiber treatment.

According to the literature, *Bifidobacteria* and *Lactobacilli*, alone or in combination, had beneficial results in patients with functional intestinal constipation and are safe for consumption.^[16–20,25]^These findings corroborate previous studies, which demonstrated a beneficial effect using strains of probiotics, alone or in combination, in patients with functional constipation or some other gastrointestinal disorder. In a trial study, Ibarra et al^[16]^ used *Bifidobacteria* for the treatment of intestinal constipation. The groups received capsules that contained the strains at 1 × 10^10^ CFU (high-dose group); capsules with the strains at 1 × 10^9^ CFU (low-dose group); and placebo capsules. The only significant difference in adequate relief of constipation was observed between the high-dose and placebo groups. The results presented in the study reported herein indicated that even with a lower dose (8 billion CFU/sachet or 3 billion CFU/sachet, but with different strains), there was an improvement in bowel function. The combination of strains can be an indication of greater effectiveness. Riezzo et al,^[17]^ in a clinical trial, employed ordinary artichokes or artichokes enriched with LP (daily dose of 2 × 10^10^ CFU) for 15 days with a daily dose of 2 × 10^10^ CFU). The trial showed a positive effect on symptoms in constipated patients after intake of probiotic-enriched artichokes. However, the number of patients (20) was small, as well as the timeframe of treatment (15 days). Lewis et al^[18]^ also used LP and *Bifidobacterium longum* to check their effectiveness ahead of irritable bowel syndrome (IBS). The authors concluded that *L. paracasei* and *B. longum* may reduce GI symptom severity and improve the psychological well-being of individuals, indicating that these strains can also be useful in the treatment of intestinal constipation. Preston et al^[19]^ reported a clinical trial for the relief of symptoms of irritable bowel syndrome, using three strains of *Lactobacillus* (LA; LC; LR). For all efficacy endpoints, improvement of 30% or more *vs*. placebo was considered clinically significant. The trial focused on evaluating several GI disorders arising from irritable bowel syndrome and not only constipation, however, it can be stated that these strains were effective for various intestinal problems, including constipation. Martínez-Martínez et al^[20]^ reported a systematic review work involving prebiotics in the treatment of elderly people. Differently, the work presented herein did not make such restriction. Those authors found that the most used strains were *Bifidobacteria* with improvement in intestinal constipation in 10% to 40% of the cases. However, the population was restricted to elderly persons and, in addition, the original study designs displayed heterogeneity. The studies, in general, corroborate the proof of the effectiveness of the LAB strains used in the present study in alleviating intestinal disorders, such as constipation.

The clinical trial reported herein had a strong adherence, with only 4 patients discontinuing the study due to losing follow-up (n = 3 participants), and 1 participant of the group that was administered with 8 billion CFU/g reporting abdominal pain. This participant reported, in the third week of use of the product, flatulence and abdominal colic. These adverse effects can occur with the use of probiotics, being reversible by simply interrupting its ingestion.^[26]^

A limitation of the study reported herein was that the results were based on self-reporting of symptoms by selected volunteers with functional constipation, as opposed to direct observation. In the research work entertained herein there was no use of a placebo control. This was yet another limitation of the study, as it may have masked the effects of probiotics in the evaluation of functional constipation.

Although there were no significant differences in the efficacy of the products, combination of strains *of Lactobacillus* and *Bifidobacterium* were well tolerated and safe for consumption. In addition, the four-week consumption of the probiotic mixtures improved digestive symptoms, especially stool quality, in adults with constipation. According to the literature, multi-strain probiotics might be more effective because of potential synergy and additive effects among the individual isolates. However, despite the availability of multi-strain probiotics, not all have shown superior benefits.^[27]^ In this study, no difference was observed between Group 1 and Group 2, indicating that a high number of probiotic strains may not be necessary.

Our findings will allow to use all the formulations as a treatment for functional intestinal constipation, since the symbiotic formulations which contained different concentrations of probiotic bacteria and the one which contained only prebiotic fibers did not produce statistical differences between them, that is, all were beneficial and had their goal achieved departing from the first week of treatment. The advantage of using only prebiotics is due to the low cost to the patient when compared to formulations integrating probiotic bacteria. Nonetheless, probiotics are live microorganisms with an expanded range of healthful activities. There is increasing evidence from the biological applications of probiotics for the maintenance and improvement of gut health, inhibition of pathogenic bacteria, and improvement of immune system and concomitant overall improvement of human health.^[27]^ Probiotics can collaborate not only with constipation, but improve overall human health, in general, due to their immune-modulating potential, their resilience against pathogen invasion of the gastrointestinal tract, or their anti-inflammatory properties.^[28]^

In conclusion, both probiotic bacteria cocktails integrating either 3 LAB strains or 8 LAB strains improved the symptoms of intestinal functional constipation by increasing both the weekly frequency of evacuation and stool quality, as early as from the first week of treatment, with sustained improvements throughout the fourth week of treatment. Treatment with these probiotic cocktails was safe and well tolerated, with only one adverse event resulting in discontinuation.

The results of this study are encouraging, but further studies are likely needed to support efficacy, safety, and durability of the effects.

## Author contributions

**Conceptualization:** Fabiana Cristina Rosa Mitelmão, Marta Maria Duarte Carvalho Vila.

**Formal analysis:** Marcus Tolentino Silva.

**Investigation:** Fabiana Cristina Rosa Mitelmão, Karin Häckel.

**Writing – original draft:** Fabiana Cristina Rosa Mitelmão, Cristiane de Cássia Bergamaschi, Victor Manuel Balcão, Marta Maria Duarte Carvalho Vila.

**Writing – review & editing:** Cristiane de Cássia Bergamaschi, Marli Gerenutti, Victor Manuel Balcão, Marta Maria Duarte Carvalho Vila.
